# Viral Enhancer Mimicry of Host Innate-Immune Promoters

**DOI:** 10.1371/journal.ppat.1003804

**Published:** 2014-02-06

**Authors:** Kai A. Kropp, Ana Angulo, Peter Ghazal

**Affiliations:** 1 Division of Pathway Medicine and Edinburgh Infectious Diseases, University of Edinburgh, Edinburgh, United Kingdom; 2 Facultad de Medicina, Institut d'Investigacions Biomèdiques August Pi i Sunyer, University of Barcelona, Barcelona, Spain; 3 SynthSys (Synthetic and Systems Biology), University of Edinburgh, Edinburgh, United Kingdom; The Fox Chase Cancer Center, United States of America

The inflammatory milieu is the natural habitat for a pathogenic infection, characterised by activity of pro-inflammatory signalling pathways and inflammatory cytokines. Viral entry rapidly activates a range of innate-immune signalling events such as the activation of Pattern Recognition Receptors (PRRs) [1]–[5]. A virus must therefore counteract intrinsic cellular and innate-immune responses to successfully complete the replication cycle. Frequently this is accomplished by encoding viral effector molecules that block these cellular responses by working as either structural or functional mimics of host target proteins [6]–[11]. Nuclear DNA viruses are dependent on the host transcriptional machinery to express the first viral genes; for example the immediate-early (IE) control elements of DNA viruses are by definition absolutely dependent on host transcription factors (TF) [12]. Therefore, these viruses are particularly hostage to their host transcriptional environment [13], [14]. Here we propose that mimicry of regulatory DNA sequences by viral regulatory regions may also provide an additional strategy to counteract at IE times of infection the innate-immune response. In this context, viral IE control elements might functionally mimic innate-immune enhancers, taking advantage of the activated immune signalling TFs for promoting viral IE gene expression.

In other words: “If you can't beat ‘em. Join ‘em.”

In exploring this possibility, we present a synopsis of the promoter-regulatory elements from seven extensively studied mammalian viruses with a DNA stage, and seven promoters representing prototypical cellular innate-immune genes. These are the SV-40 early enhancer, the E1A enhancer of HAdV5, the long terminal repeat (LTR) of HIV-1, the E6/7 long control region (LCR) of both HPV-16 and HPV-18, the major IE (MIE) enhancer of HCMV, and the enhancer-1 (Eh-1) regulatory region of HBV for viral sequences, and the enhancer regions of human IFNB1, IFNG, TNF, IRF1, IL8, IL12B, and IL1B for host sequences. First, we consider similarities between the primary sequence structures of the enhancers. Second, we present arguments for convergent evolution and structural flexibility inherent to enhancer sequences. Third, we discuss functional features and regulatory hallmarks that may be used to define viral enhancer mimicry of cellular immune enhancers.

## Do Viral and Cellular Enhancers Display Any Primary Sequence Similarity?

**Figure 1:**
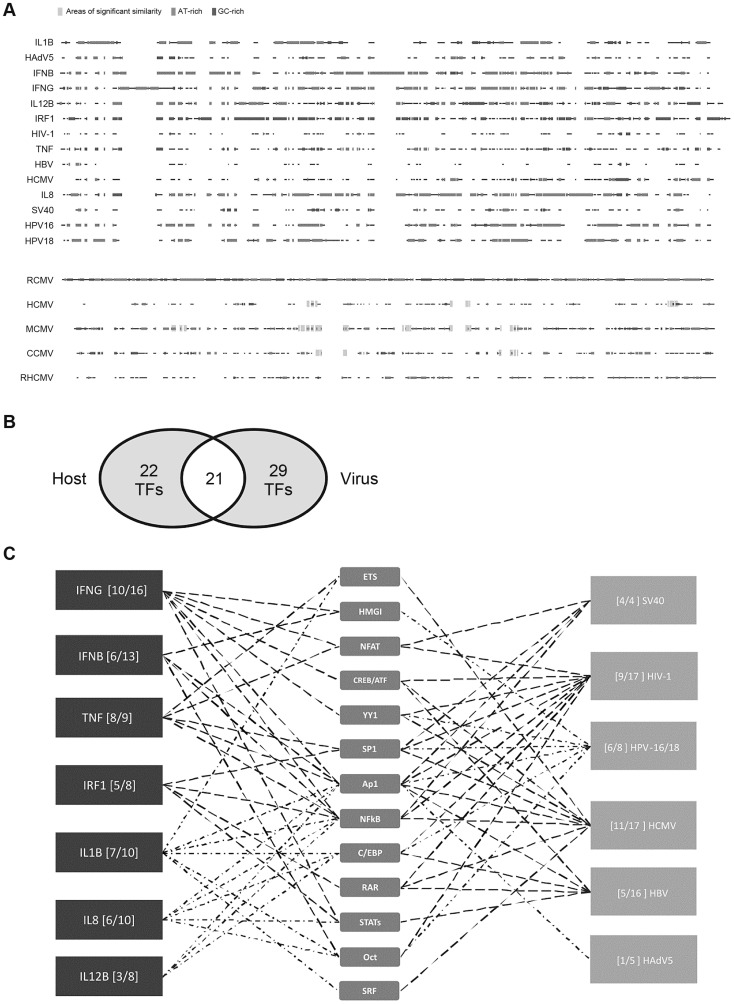
Comparison of host innate-immune and viral regulatory regions. A) Multi-way alignment of analysed enhancer sequences shows no sequence similarity. Narrow grey boxes mark AT-rich stretches and dark grey boxes mark GC-rich stretches. Overall, sequence similarity was too low to produce a phylogenetic tree. To analyse sequence similarity within one family of viruses, we compared the major immediate-early enhancer region of rat-CMV (RCMV) with those of human (HCMV), murine (MCMV), chimpanzee (CCMV) and rhesus (RHCMV) cytomegalovirus. Small stretches of sequences similarity to the RCMV sequence are indicated by wide light grey boxes (similarity >80%). B) Venn diagram of 72 TFs identified in our literature search to interact with the analysed regulatory sequences. Detailed SBGN diagrams of all elements and interactions can be found at [46]–[52] except for TNF [57]. C) Simplified summary of transcription factor families shared between analysed innate-immune regulatory regions and viral control elements. For simplification interactions with members belonging to a family of TFs are represented by only one symbol (e.g., p50, p65, and RelA interactions are all represented by the “NFkB” symbol). The summary was produced in the “MSc by research in genomics and pathway biology” project by literature review. Digits in brackets indicate the number of shared interactions (left of dash) and total number of interactions for the specific enhancer (right of dash). TFs that are more highly connected with viral and host elements were placed toward the centre.

To investigate if there is any similarity of primary sequences and therefore structural mimicry between the selected viral and cellular enhancers, we used the BLAST tool to compare the sequences against each other (Table 1) and applied an exhaustive pairwise multi-way alignment (CloneManager suite 7.0) to search for similarities in this group of sequences (Figure 1A). While multi-way alignment of the various selected viral and cellular promoter-regulatory regions (Figure 1A, top panel) reveals a lack of extended primary sequence homology, the pairwise BLAST comparison showed that small islands of sequence identity or high similarity are present (Table 1). We randomly compared some of these short sequence motifs with the JASPAR CORE (Vertebrae) database [15] and found that all checked motifs have similarities with consensus binding motifs for TFs (e.g., AP1, SP1, YY1, or RelA with relative scores of >0.8). This finding raises the question of whether there might be functional similarity. We therefore consider in the next section how convergent evolution of viral enhancers may have resulted in functional mimicry of the transcription control elements of innate-immune genes, providing a co-opting strategy for immune evasion.

**Table 1:** Summary of pairwise sequence comparison.

Viral		HCMV	HIV-1	HPV-16	HPV-18	HBV	SV-40	HAdV5
	Selected enhancer region	Major immediate-early enhancer (MIE)	Long terminal repeat (LTR)	E6/7 Long control region (LCR)	E6/7 Long control region (LCR)	Enhancer-1 regulatory region (Eh-1)	Early enhancer (SV-40)	E1A enhancer
	Number of small islands of high similarity (BLAST)	IFNG (2), SV-40 (5), HPV-18 (1)	IL1B (2), IL12B (1)	IL1B (1), IL8 (1), IL12B (1), HAdV5 (5), HPV-18 (3)	IFNG (1), HCMV (1), IL12B (1), HPV-16 (4)	IL12B (1)	HCMV (2)	IFNG (1), IFNB1 (2), IL8 (1), IL12B (1), HPV-16 (1)
Host		IFNG	IFNB1	IL1B	IL12B	TNF	IRF1	IL8
	Selected enhancer region	−1 kb region	−1 kb region	−1 kb region	−1 kb region	−650 bp core enhancer region	−1 kb region	−1 kb region
	Number of small islands of high similarity (BLAST)	HCMV (2), IFNB1 (1), IL8 (1), IL1B (1), IRF1 (1), IL12B (1), HAdV5 (1), HP-18 (1)	IFNG (1), IL8 (2), HAdV5 (2)	IFNG (1), IL8 (2), HPV-16 (1), HIV-1 (2)	IFNG (1), IRF1 (1), HAdV5 (1), HBV (1), HPV-16 (1), HPV-18 (2), HIV-1 (1), TNF (4)	IL8 (1), IRF1 (1), IL12B (2)	IFNG (1), IL12B (1), TNF (1)	IFNG (1), IFNB1 (3), IL1B (2), TNF (1), HAdV5 (1), HPV-16 (1)

Pairwise comparison of analysed enhancer sequences and number of similarity islands identified by BLAST (blastn) alignment. Each regulatory region was used as a reference sequence and compared to all other sequence elements. The number of sequence motifs with high similarity produced from this analysis are given in parentheses; comparisons that produced no significant similarities are not shown.

## Could Viral Regulatory Regions Evolve as Functional Mimics of Innate-Immune Enhancers without Extensive Sequence Similarity?

There are two principal genetic mechanisms that could lead to viral mimicry of host enhancers, horizontal transfer of cellular sequences to viral genomes or genetic drift of viral sequences. The first possibility, acquisition of cellular sequences through horizontal sequence transfer, could arise through illegitimate recombination with host DNA, for example by retro-transposition of non-coding RNA transcripts, resulting in the virus hijacking host transcription control sequences. If this were the general case, we would, however, expect significant structural similarity, which we did not find in our analysis. Alternatively, but not mutually exclusive from horizontal transfer, viral enhancer mimics could arise through neutral evolution and genetic drift by sequence duplication or accumulation of point mutations. Duplicated sequence features are hallmarks for many viral and cellular enhancers [16]–[24]. For instance, deletion or loss of enhancer sequences in SV40 and JC polyomavirus promotes restoration of enhancer function through duplication of flanking sequences [25]–[28]. A third possibility is the accumulation of point mutations in enhancer sequences and subsequent fixation [29]. It has recently been described for a wide range of species that evolution of host-cell transcriptional control can occur in relatively short time spans and is mainly driven by the rapid and flexible emergence or loss of binding motifs rather than by evolution of the TF proteins themselves [30]–[36]. The described mechanisms of rapid enhancer evolution argue that viral enhancers could acquire functionality that mimics innate-immune enhancers without any extensive sequence homology, and this is consistent with the comparison of cellular and viral enhancers shown in Figure 1A. This possibility is underscored by the fact that promoter sequences seem to be poorly conserved even among members within a virus-family yet share many of the same regulatory elements [37]. For example the MIE enhancers of cytomegaloviruses show low levels of primary sequence similarity between the different species strains (Figure 1A, lower panel). Despite these differences, functionality of the enhancers is conserved between hosts for different CMV species strains, e.g., the human CMV enhancer can functionally complement deletion of the murine CMV enhancer [38] and human CMV enhancer sequences recapitulate *in vivo* biological sites of infection in species from mice to zebra fish [39]–[41].

## What Features Would Classify a Viral Enhancer as an Innate-Immune Enhancer Mimic?

Since our work and that of others discussed so far indicates that viral enhancers are functional rather than structural mimics of host innate-immune enhancers, we suggest four principal hallmarks of functional enhancer mimicry. These are: 1) shared TF interactions independent of sequence structure, 2) similar kinetics of gene induction between cellular innate-immune and viral IE genes, 3) positive responsiveness to immune-stimulatory ligands, and 4) susceptibility to inhibition of inflammatory signalling. In the following section we briefly discuss these hallmarks.

### Shared Transcription Factor Interactions

The human genome encodes an estimated 1,700 to 1,900 TFs, with 1,391 representing high-confidence candidates [42]. These proteins represent an ample resource for viruses to harness. To probe, in more detail, the TF usage of the 14 viral and innate-immune enhancers selected (Table 1), we constructed unambiguous diagrams [43]–[45] of known TF interactions—available as an online resource on Figshare [46]–[52]. Using this approach we identified 72 interactions (Table 2) between the selected host and viral regulatory regions and host TFs. Of the 72 interactions identified, 43 were described for cellular enhancers and 50 for viral enhancers and 21 interactions (49% and 42% respectively) are shared among innate-immune enhancers and viral enhancers (Figure 1B). Annotation of this dataset using the BioMART tool (v0.7, ENSEMBL release v72) identified 31 TFs associated with “regulation of immune processes” [GO:0002376] in our 72 identified interactions. Notably, the extent to which the distinct viral enhancers share factorswith the innate-immune genes varies (Figure 1C). This may be explained by the different physiological roles of the innate-immune genes and lifestyles of the selected viruses. Among the viruses, HCMV and HIV-1 enhancers show the largest TF overlap in total numbers of interactions with the innate-immune genes. In summary, we identified a substantial overlap in TF interactions between host and viral regulatory regions.

**Table 2:** List of identified interactions for the selected viral and host enhancers.

TF Name	Entrez Gene ID	Protein Family	TF Name	Entrez Gene ID	Protein Family
NFKB1 (p50)	4790	NFkB	MYOF	26509	Ferlin
RelA (p65)	5970	NFkB	HSF1	3297	HSF
RelC	5966	NFkB	ELK1	2002	ETS
NFkB2 (p52)	4791	NFkB	SRF	6722	SRF
C/EBP	N/A (generic)	C/EBP	RAR	5914	Nuclear hormone receptor
CREB1	1385	bZIP	RXR	6256	Nuclear hormone receptor
ATF1	466	AP	ETS2	2114	ETS
ATF2	1386	AP	GAP12	Unspecified	Unspecified
AP1/Jun	3725	AP	NRF (NKRF)	55922	N/A
FOS	2353	AP	NF1	4763	Nuclear hormone receptor
SP1	6667	C2H2-zinc finger	GRE/NR3C1	N/A (generic)	Nuclear hormone receptor
SPI1	6688	ETS	AP2	7020	AP
HMGI(Y)	3159	HMG	AP3	Unspecified	Unspecified
OCT 1	5451	OCT/POU	USF1	7391	Helix-loop-helix leucine zipper
OCT 2	5452	OCT/POU	TFE3	7030	MiT/TFE
IRF1	3659	IRF	LEF1	51176	TCF/LEF
IRF2	3660	IRF	ETS1	2113	ETS
IRF3	3661	IRF	OTK18	7728	Krueppel C2H2-zinc finger
IRF7	3665	IRF	E2F1	1869	EF
STAT1	6772	STAT	BCL3	602	N/A
STAT2	6773	STAT	SP3	6670	C2H2-zinc finger
STAT3	6774	STAT	ERF	2077	ETS
STAT4	6775	STAT	GFI1	2672	C2H2-zinc finger
NFATp/NFATc	4773/511224	NFAT	CUX1	1523	N/A
NFIL6	1051	bZIP	E1A	6870514	Adenoviridae E1A protein
YY1	7528	YY	E4F1	1877	EF
TBX21	30009	T-BOX	TAF1	6872	TAF1
EOMES	8320	T-BOX	HBS1L	10767	N/A
PPAR	N/A (generic)	Nuclear hormone receptor	HNF1	6927	Hepatic nuclear factor
PPARG/PROX1	5468/5629	Nuclear hormone receptor	HNF3	2305	Hepatic nuclear factor
SMAD3	4088	SMAD	HNF4	3172	Hepatic nuclear factor
RUNX3	864	N/A	RFX1	5989	RFX
PRDM1/PRDI BF1	639	C2H2-zinc finger	PX	944566	Orthohepadnavirus protein X
HIVEP2/PRDII BF1	3097	C2H2-zinc finger	C-abl	25	Tyr protein kinase family
HIVEP1	3096	C2H2-zinc finger	NR2F1/COUP-TF	7025	Nuclear hormone receptor
NREBP	6651	N/A	PEF1	553115	Penta-EF-hand protein

### Comparable Expression Kinetics

It is noteworthy that host immediate-early response genes and viral immediate-early genes are, by definition, identified by the same criterion, namely that their expression is independent of newly synthesised proteins [12], [53], [54]. Upon infection of permissive cells, viral promoters are activated within the first hour of infection. This follows a typical expression profile with a peak between 2–6 h followed by reduced expression levels. This expression pattern has parallels with the temporal expression of host innate-immune genes, e.g., IFNB1, IL6, or TNF that are rapidly induced after PRR activation [55]–[58]. Most notably, it has recently been demonstrated in a genome-wide transcriptome study with murine CMV that the mRNA synthesis rate of viral IE transcripts is rapidly induced and subsequently strongly downregulated, following the expression kinetic profile for many innate immune genes in this dataset [59].

### Response to Immune-Stimulatory Ligands

A corollary of viral enhancer mimicry of innate-immune regulatory functions is that the viral promoters/enhancers should be activated by the same signalling events as innate-immune genes. This implies that events during the infection process that trigger “antiviral” signalling cascades actually facilitate the initial viral transcription. In this context, it has been shown that activation of TLRs by LPS and CpG [60], [61] increases activity in isolated HCMV-enhancer and HIV-LTR–driven reporter constructs [62]–[64]. This also seems to apply in the context of viral infection since cytokine signalling stimulates HBV gene expression [65] and HIV needs TLR-8 signalling in specific cell types for replication [66]. It is also notable that all of the viral control regions examined here have been shown to interact with AP-1 (Figure 1C). While AP-1 is not exclusively associated with innate-immune signalling, it can be activated by TLR signalling via MAPK-activation or by cAMP-related signalling during infection [67], [68] and subsequently also binds to innate-immune enhancers. Taken together, these examples indicate that so called “antiviral” processes have the potential to facilitate viral IE gene expression and replication. In the future, their importance and potentially proviral role should be examined in viral infection models.

### Responsiveness to Negative Feedback Control

Immune signalling pathways are tightly regulated by negative feedback with the inhibitors of signalling activity acting in a matter of minutes to hours [69], [70]. Thus, innate-immune negative feedback loops should also inhibit viral gene expression and may play a role in viral latency. This hallmark of viral enhancer mimicry might prove the most challenging for scientific investigation. Interference with negative feedback regulators before infection may lead to an exacerbated immune response, either inducing an elevated antiviral state in the cell before the experimental infection or driving it into apoptosis. Still, proving that this hallmark is applicable to viral infections might provide new drug targets to inhibit viral infections. While, to our knowledge, no direct effects of negative regulators of inflammatory signalling on viral gene expression have been reported so far, it has been shown that anti-inflammatory drugs and chemical inhibitors of pro-inflammatory signalling, expected to increase viral replication, actually can inhibit viral gene expression and replication of HCMV, HBV, and HIV-1 [67], [68], [71]–[74].

## Concluding Remarks

TFs activating innate-immune genes are regulated by PRR signalling that cannot be efficiently inhibited by viruses as their activation occurs during the viral entry process. Mimicking an innate-immune enhancer therefore has the advantage that TFs, already activated by the viral entry process, can be directly utilised in a time restricted manner to ensure viral gene expression at IE times. We hope this opinion opens debate and provides new insights for either reexamination or future-based investigations toward understanding viral gene activation and latency. Indeed we believe that the principle of viruses co-opting host-innate regulatory signals has broad implications toward understanding the biological role of viral enhancers, in acute and latent viral infections, and prospective host-directed antiviral therapeutic and vaccine strategies.
